# Performance of “VIKIA Malaria Ag Pf/Pan” (IMACCESS®), a new malaria rapid diagnostic test for detection of symptomatic malaria infections

**DOI:** 10.1186/1475-2875-11-295

**Published:** 2012-08-24

**Authors:** Monidarin Chou, Saorin Kim, Nimol Khim, Sophy Chy, Sarorn Sum, Dany Dourng, Lydie Canier, Chea Nguon, Didier Ménard

**Affiliations:** 1 Faculty of Pharmacy, Université des Sciences de la Santé, Phnom Penh, Cambodia; 2 Malaria Molecular Epidemiology Unit, Institut Pasteur du Cambodge, Phnom Penh, Cambodia; 3 National Center for Parasitology, Entomology and Malaria Control, Phnom Penh, Cambodia

## Abstract

**Background:**

Recently, IMACCESS**®** developed a new malaria test (VIKIA Malaria Ag Pf/Pan™), based on the detection of falciparum malaria (HRP-2) and non-falciparum malaria (aldolase).

**Methods:**

The performance of this new malaria rapid diagnostic test (RDT) was assessed using 1,000 febrile patients seeking malaria treatment in four health centres in Cambodia from August to December 2011. The results of the VIKIA Malaria Ag Pf/Pan were compared with those obtained by microscopy, the CareStart Malaria™ RDT (AccessBio®) which is currently used in Cambodia, and real-time PCR (as “gold standard”).

**Results:**

The best performances of the VIKIA Malaria Ag Pf/Pan™ test for detection of both *Plasmodium falciparum* and non-*P. falciparum* were with 20–30 min reading times (sensitivity of 93.4% for *P. falciparum* and 82.8% for non-*P. falciparum* and specificity of 98.6% for *P. falciparum* and 98.9% for non-*P. falciparum*) and were similar to those for the CareStart Malaria™ test.

**Conclusions:**

This new RDT performs similarly well as other commercially available tests (especially the CareStart Malaria™ test, used as comparator), and conforms to the World Health Organization’s recommendations for RDT performance. It is a good alternative tool for the diagnosis of malaria in endemic areas.

## Background

Now that artemisinin derivatives in combination with partner drugs (artemisinin combination therapy (ACT)) are being used, parasitological confirmation has become essential before treatment in routine malaria case management, in most countries endemic for malaria [1,2]. This ensures that anti-malarial drugs are only administered to patients who need them, thereby limiting the unnecessary use of inappropriate treatments. It also minimizes the selection and spread of drug-resistant *Plasmodium falciparum* parasites [3], particularly important in areas where multidrug resistance is prevalent, such as Southeast Asia [4-7].

Microscopic examination of blood films still remains the gold standard for malaria diagnosis despite the requirement for high-quality microscopes and equipment, and for qualified personnel [8]. However, over the past two decades, rapid diagnostic tests (RDTs) for malaria have been developed for use in any situation where the only realistic alternative was clinical diagnosis of malaria, for example, at community level (e g, Village Malaria Workers’ strategy in Cambodia). These diagnostic tests are fast and easy to use, and do not require electricity or complex equipment [9]. RDTs are now available from around 60 manufacturers [10]. This profusion of suppliers and the variable quality of RDT products marketed have made it difficult for the policy makers of national malaria control programmes to determine which tests are the most suitable [11]. To address this issue, a global evaluation programme to guide RDT procurement (product testing) and assure RDT performance before and during use in the field (lot testing) was launched in 2002 by the WHO Regional Office of the Western Pacific (WPRO), in collaboration with the WHO/Global Malaria Programme, WHO/GMP), the UNICEF/UNDP/World Bank/WHO Special Programme for Research and Training in Tropical Diseases (TDR), the Foundation for Innovative New Diagnostics (FIND), the Centers for Disease Control and Prevention (CDC, Atlanta) and numerous other partners [12].

The malaria RDTs available are all based on the same principle and like other lateral flow immune-chromatographic tests, there are various formats (dipstick, plastic cassette or card). They contain antibodies conjugated to colloidal gold particles, which bind specifically with parasite antigens. Some of them are specific for *P. falciparum* and detect the histidine-rich-2 protein (HRP-2). Others combine HRP-2 detection with the detection of antigens common to all species, such as lactate dehydrogenase or aldolase (combo RDTs). Combo RDTs can diagnose infections by *P. falciparum* or by non-*P. falciparum* malaria parasites (*Plasmodium vivax, Plasmodium ovale* and *Plasmodium malariae*) [13].

IMACCESS^©^ is a company which aims to address public health requirements by developing reliable diagnostic tools responding to the constraints associated with use in the field for populations in developing countries at affordable prices [14]. The company recently developed VIKIA Malaria Ag Pf/Pan™, a new malaria test. This new RDT, based on the same technology as other tests, allows detection of falciparum malaria (HRP-2) and non-falciparum malaria (aldolase).

In this context, the objective of the study presented here was to assess the performances of this new immunochromatographic test by following a conventional field evaluation design including microscopic examination (thick and thin blood film) and molecular detection (real-time PCR) as gold standard methods for malaria diagnosis of febrile, malaria-suspected patients seen at health centre level. In addition, the VIKIA Malaria Ag Pf/Pan™ test was also compared to another RDT widely used in Cambodia and elsewhere (CareStart Malaria™, AccessBio®).

## Methods

### Rapid diagnostic tests

**Figure 1:**
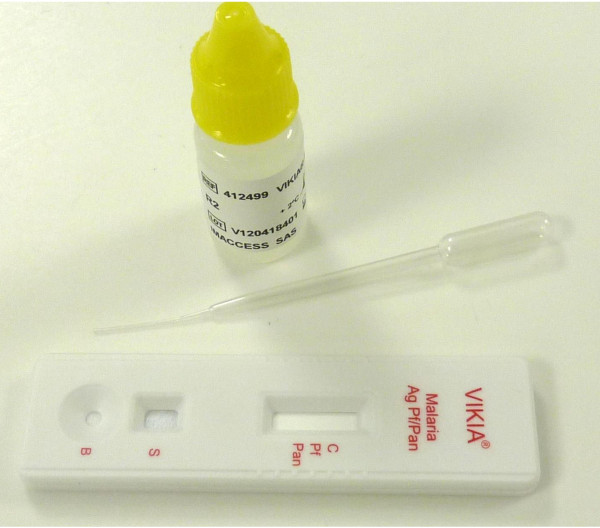
Design of the VIKIA Malaria Ag Pf/Pan™ test.

The VIKIA Malaria Ag Pf/Pan™ test kit (IMACCESS^©^, Ref. 41 2499, Lot Number: V110727601, Lyon, France), containing one package insert, 25 test cassettes in individual sealed pouches with a disposable specimen pipette and a dessicant and one vial of running lysis buffer, was used according to the manufacturer’s instructions. Briefly, a 5 μL aliquot of blood drawn from patients was added to the sample well. Five drops of lysis buffer were then dispensed into the buffer well (Figure 1). Results from the test were interpreted at several times (readings after 10, 15, 20, 30 and 60 min). In parallel, the CareStart Malaria™ test (Access Bio^©^, Ref. G0131, Lot Number: J191R, New Jersey, USA) was also performed according to the manufacturer’s instructions and used to diagnose patients as malaria infected.

### Study population

**Figure 2:**
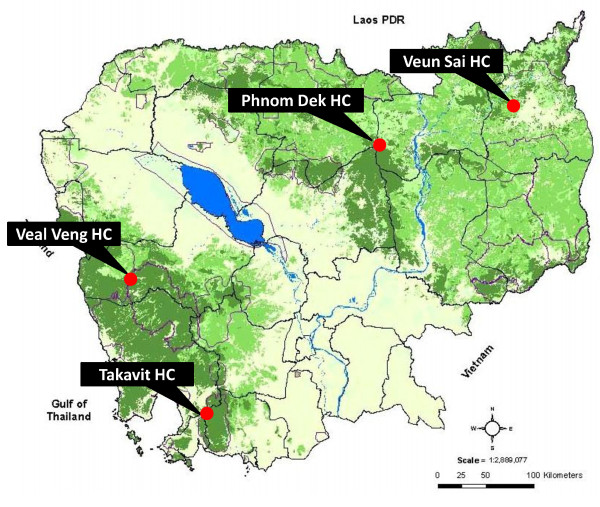
Map of Cambodia with the locations of the four selected health centres indicated.

Four different sites in Cambodia, all members of the anti-malarial drug-resistance network (collaboration between the Ministry of Health and the Institut Pasteur du Cambodge) were selected (Figure 2): Veal Veng health centre (in Pursat province), Veun Sai health centre (in Rattanakiri province), Phnom Dek health centre (in Preah Vihear province) and Takavit health centre (in Sihanoukville province).

Malaria remains a public health problem at these sites, and is mainly linked to the forests. Forest villagers in the eastern and northern provinces are at high risk of malaria, whereas elsewhere in the country malaria is an occupational disease with particular groups being at high risk, including forestry workers, new settlers and mobile/migrant populations moving into forested areas, and soldiers and their families serving in the forests. The prevalence is between 15% and 40% in villages near or in forested areas, and between 0% and 3% in the plains and rice field areas. The five human *Plasmodium* species all co-exist in the Cambodia and *P. falciparum* is the most frequent cause of malaria infections (prevalence ~70%). The distributions of *Plasmodium* species have been changing over recent years, with a particularly significant increase in the incidence of *P. vivax* malaria cases (from 8% in 2000 to 23% in 2007). In low transmission areas, a proportion of infections that are *P. vivax* is commonly up to 50% [15-19].

In the four sites, patients with clinical symptoms of malaria (fever ≥37.5°C) and with a prescription for malaria diagnosis from a health care worker, agreeing to be enrolled in the study (by signing the consent form), were included. Pregnant women and patients with signs of severe and complicated *P. falciparum* malaria according to the definition of the World Health Organization (2001) were excluded [20]. The study protocol was reviewed and approved by the Ethics Committee of the Cambodian Ministry of Health (No. 075 NECHR, 21 June, 2011).

### Sample collection

Blood samples were collected by finger-prick and used for malaria testing (both RDTs were tested in parallel by two health staff blinded to each other). In addition, two drops of blood were spotted onto Whatman 3 M filter paper™ and individually stored in an envelope and a plastic bag. Slides for thick and thin microscopic examination were also prepared, and stored in appropriate boxes. Clinical and biological information were recorded on an anonymous form to ensure patient confidentiality. Filter papers and slide boxes were sent to the Institut Pasteur du Cambodge (IPC) in Phnom Penh every week.

### Laboratory procedures

At IPC, thick and thin blood smears were stained with 3% Giemsa for 30 min and analysed by light microscopy by two experienced technicians without reference to RDT results. A minimum of 200 consecutive fields was checked in the thick blood film before classifying a slide as negative. Parasites in thick blood films were counted against 200 white blood cells. The parasite density was estimated assuming 8,000 white blood cells/μL of blood. One in 10 slides selected at random, and all slides corresponding to discordance between the first two readings, were read a third time by a third expert microscopist.

DNA was extracted from blood spots with Instagene Matrix resin™ (Bio-Rad^©^, Marnes la Coquette, France), according to the manufacturer’s instructions. Molecular detection and identification of *Plasmodium* parasites involved two steps: *Plasmodium* was detected by a “screening real-time PCR” with primers targeting the *Plasmodium* cytochrome b gene. Secondly, DNA samples identified as positive for *Plasmodium* were analysed for malaria species by PCR with several pairs of specific primers (*P. falciparum, P. vivax/ Plasmodium knowlesi, P. malariae* and *P. ovale*) targeting the same gene (Table 1). Molecular analyses were performed by technicians blind to the results of microscopic and RDT diagnoses.

**Table 1:** **Primers sequences and real-time PCR conditions used to detect *Plasmodium *species, Cambodia, 2011**

**Real-time PCR name**		**Primer name**	**Sequence (5'-3')**	**Master mix**	**Assay parameters**	**Melt Parameters**	**T° Melt peak**
**"Screening"**		RTPCRScreening2_F	TGGAGTGGATGGTGTTTTAGA	Hot FirePol EvaGreen qPCR Mix Solis Biodyne 1X (#08- 24-00020), Primers 250 nM, 5 μl DNA template, total volume 20 μl	95°C-15 min. 45 cycles: 95°C-15 sec./60°C-20 sec./ 72°C-20 sec. 95°C-2 min. 68°C-2 min	From 68 to 90°C, increment 0.2°C for 0.05 sec.	76.2 - 78.8°C
		RTPCRScreening2_R	TTGCACCCCAATARCTCATTT				
***Plasmodium *sp. identification**	Primary PCR	RTPCRScreening2_F	TGGAGTGGATGGTGTTTTAGA	Hot FirePol DNA Pol. Solis BioDyne 1.25 U (#01-02-01000), dNTP 200 μM, MgCl2 2.5 mM, Primers 250 nM, 5 μl DNA template. Total volume 20 μl.	94°C −15 min. 20 cycles: 94°C-30 sec/ 58°C -1 min/ 72°C-1 min3. 72°C-10 min.	N/A	N/A
		RTPCRSreening3_R	ACCCTAAAGGATTTGTGCTACC				
	Pf real-time PCR	Pf_RTPCR_F	ATGGATATCTGGATTGATTTTA TTTATGA	Hot FirePol EvaGreen HRM Mix Solis Biodyne 1X (#08- 33-00001), Primers 250 nM, 5 μl template Primary PCR products 1:1000, total volume 20 μl	95°C-15 min. 40 cycles: 95°C-10 sec./62°C-20 sec./ 72°C-25 sec. 95°C-1 min. 40°C-1 min	From 65 to 90°C, increment 0.2°C for 0.05 sec.	78.8-79.6°C
		Pf_RTCPR_R	TCCTCCACATATCCAAATTAC TGC				
	Pv real-time PCR	Pv_RTPCR_F	TGCTACAGGTGCATCTCTTG TATTC				75.2-76°C
		Pv_RTPCR_R	ATTTGTCCCCAAGGTAAAACG				
	Pm real- time PCR	Pm_RTPCR_F	ACAGGTGCATCACTTGTATTTTTTC				75.8-76.2°C
		Pm_RTPCR_R	TGCTGGAATTGAAGATAATAAATT AGTAATAACT				
	Po real- time PCR	Po_RTPCR_F	GTTATATGGTTATGTGGAGGATA TACTGTT				73.4-74.2°C
		Po_RTPCR_R	CGAATGGAAGAATAAAATGTAG TACG				

### Data analysis

Data were entered, processed, and analysed using Microsoft Excel 2010 software. The chi-squared test was used to compare the performances of the different diagnostic methods used (real-time PCR, microscopy and RDTs). *P-*values <0.05 indicated statistically significant differences. For sensitivity and specificity, RDT results were compared with microscopy and real-time PCR results. Sensitivity was calculated as the proportion of positive test results obtained among samples containing malaria parasites as identified by microscopy or real-time PCR. Specificity was calculated as the proportion of negative test results among samples scoring negative by thick blood film or real-time PCR tests. Positive and negative predictive values were the proportion of true-positive results among all positive samples and the proportion of true negative results among all negative samples, respectively.

## Results

From August to December 2011, 1,000 patients (Preah Vihear, N = 300, Veal Veng, N = 250, Ratanakiri, N = 300 and Takavit, N = 150) with an age range of one to 70 years (mean ± SD age = 22.4 ± 12.9 years; 7.0% <5 years of age, 18.0% between five and 14 years of age, and 75.0% >15 years of age) were recruited. The male/female ratio was 2.1/1. The mean ± SD axillary temperature was 38.1 ± 0.9°C (range = 37.5–41.5°C) and the mean ± SD parasitaemia density was 13,455 ± 52,694 parasites/μL (range = 20–582,500 parasites/μL).

Microscopy results showed that 389 (38.9%) of the 1,000 patients had malaria. *Plasmodium falciparum* was present in 39.5%, *P. vivax* in 58.8% and both *P. falciparum* and *P. vivax* (mixed infections) in 1.5% of the positive specimens. A total of 92 discordant results (9.2%) were found between microscopy and real-time PCR (Table 2). Some 32 cases (3.2%) classified as negative by microscopy were positive by real-time PCR (sub-microscopic parasitaemia: six *P. falciparum*, six mixed *P. falciparum/P. vivax* and 21 *P. vivax*). In addition, 59 samples were misclassified by microscopy: 54 were classified as single infections by microscopy (43 *P. falciparum*, 11 *P. vivax*) but found to be mixed infections by real-time PCR (52 mixed *P. falciparum/P. vivax* and two mixed *P. vivax/P. malariae);* three cases classified as mixed *P. falciparum/P. vivax* infections were identified as single *P. falciparum* infections by real-time PCR; two cases classified as single *P. vivax* infections were found to be single *P. falciparum* infections by real-time PCR.

**Table 2:** Comparison of real-time PCR and microscopy results for 1,000 patients tested for malaria at health centres, Cambodia, 2011

**PCR results**	**Microscopy results**				**Total**
	**Negative**	***P. falciparum***	***P. falciparum/ P. vivax***	***P. vivax***	
Negative	578	0	0	0	578
*P. falciparum*	6	110	3	2	121
*P. falciparum/ P. vivax*	6	43	4	9	62
*P. vivax*	21	0	0	216	237
*P. vivax/ P malariae*	0	0	0	2	2
Total	611	153	7	229	1000

**Figure 3:**
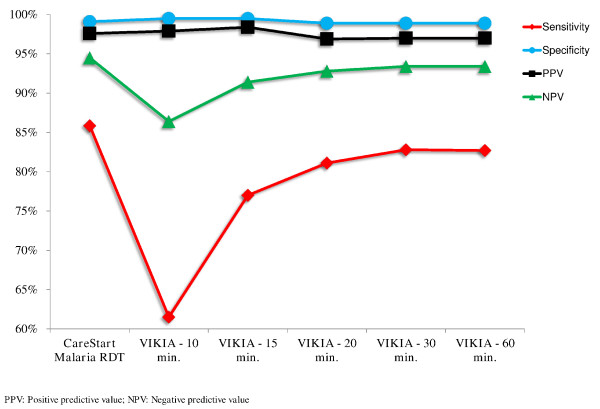
**Comparative representation of the diagnostic performances of the CareStart Malaria™ test and the VIKIA Malaria Ag Pf/Pan™ test at several reading times (10, 15, 20, 30 and 60 minutes) for detection of*Plasmodium spp***. **in field study patients, Cambodia, 2011**.

**Figure 4:**
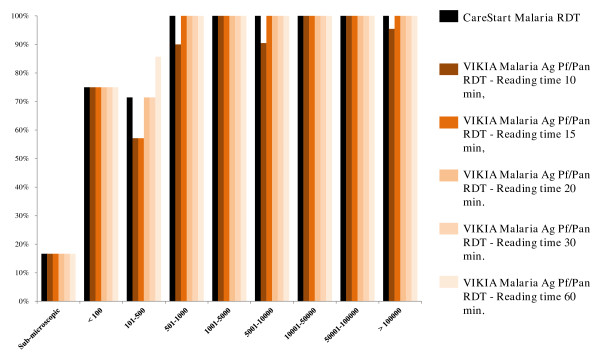
**Comparative representation of the sensitivities of the CareStart Malaria™ test and the VIKIA Malaria Ag Pf/Pan™ test at several reading times (10, 15, 20, 30 and 60 minutes) for different levels of *Plasmodium *spp. parasitaemia.** Results for *Plasmodium falciparum* infections, Cambodia, 2011.

**Figure 5:**
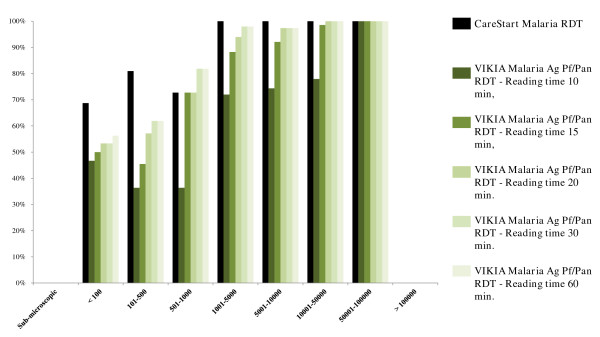
**Comparative representation of the sensitivities of the CareStart Malaria™ test and the VIKIA Malaria Ag Pf/Pan™ test at several reading times (10, 15, 20, 30 and 60 minutes) for different levels of *Plasmodium *spp. parasitaemia.** Results for non-*P. falciparum* infections, Cambodia, 2011.

Details concerning patients positive for *Plasmodium* spp. by real-time PCR, microscopy, CareStart Malaria™ and VIKIA Malaria Ag Pf/Pan™ tests at several reading times (10, 15, 20, 30 and 60 min) are shown in Table 3. Frequencies of false positives, false negatives and misclassified results obtained from RDTs with reference to microscopy and PCR results (including non-interpretable data; Table 4) indicated that the best reading times were 20–30 min for the VIKIA Malaria Ag Pf/Pan™ test. Diagnostic performances of both RDTs are shown in Table 5 and Figure 3. Sensitivities for different levels of *Plasmodium* spp. parasitaemia are summarized in Table 6, Figure 4 for *P. falciparum* infections and Figure 5 for non-*P. falciparum* infections.

**Table 3:** **Patients scoring positive for *Plasmodium *spp. by the reference method (microscopy/PCR), CareStart Malaria™ test and VIKIA Malaria Ag Pf/Pan™ test at several reading times (10, 15, 20, 30 and 60 minutes), Cambodia, 2011.**

**RDT results**		**Microscopy and real-time PCR results**					**Total**
		**Negative**	***Pf***	***Pf/Pv***	***Pv***	***Pv/Pm***	
		**578**	**121**	**62**	**237**	**2**	**1000**
		**(57.8%)**	**(12.1%)**	**(6.2%)**	**(23.7%)**	**(0.2%)**	**(100%)**
CareStart Malaria RDT	Negative	570	8	8	33	0	619
	*Pf & Pf/Non-Pf*	3	113	46	6	0	168
	*Non-Pf*	5	0	8	198	2	213
	Non interpretable	0	0	0	0	0	0
VIKIA Malaria Ag Pf/Pan RDT - Reading time 10 min.	Negative	574	12	16	89	1	692
	*Pf & Pf/Non-Pf*	1	104	41	3	0	149
	*Non-Pf*	3	3	5	143	1	155
	Non interpretable	0	2	0	2	0	4
VIKIA Malaria Ag Pf/Pan RDT - Reading time 15 min.	Negative	573	9	9	54	0	645
	*Pf & Pf/Non-Pf*	2	111	46	4	0	163
	*Non-Pf*	3	1	7	179	2	192
	Non interpretable	0	0	0	0	0	0
VIKIA Malaria Ag Pf/Pan RDT - Reading time 20 min.	Negative	570	8	8	44	0	630
	*Pf & Pf/Non-Pf*	2	113	46	6	0	167
	*Non-Pf*	6	0	8	187	2	203
	Non interpretable	0	0	0	0	0	0
VIKIA Malaria Ag Pf/Pan RDT - Reading time 30 min.	Negative	570	8	8	40	0	626
	*Pf & Pf/Non-Pf*	2	113	46	6	0	167
	*Non-Pf*	6	0	8	191	2	207
	Non interpretable	0	0	0	0	0	0
VIKIA Malaria Ag Pf/Pan RDT - Reading time 60 min.	Negative	567	7	8	40	0	622
	*Pf & Pf/Non-Pf*	5	114	46	7	0	172
	*Non-Pf*	6	0	8	190	2	206
	Non interpretable	0	0	0	0	0	0

Pf: P. falciparum; Pv: Plasmodium vivax; Pm: Plasmodium malariae; Non-Pf: Plasmodium non-falciparum.

**Table 4:** Frequencies of false positive, false negative and misclassified results obtained from RDTs with reference to microscopy and PCR results (including non-interpretable data), Cambodia, 2011

**RDTs**		**Frequency of**				**Total**
		**False positive**	**False negative**	**Misclassified**	**Non-interpretable**	
CareStart Malaria RDT		0.8	4.9	1.4	0	7.1
VIKIA Malaria Ag Pf/Pan RDT	Reading time 10 min.	0.4	11.8	1.1	0.4	13.7
	Reading time 15 min.	0.5	7.2	1.2	0	8.9
	Reading time 20 min.	0.8	6.0	1.4	0	8.2
	Reading time 30 min.	0.8	5.6	1.4	0	7.8
	Reading time 60 min.	1.1	5.5	1.5	0	8.1

**Table 5:** **Diagnostic performances of the CareStart Malaria™ test and the VIKIA Malaria Ag Pf/Pan™ test at several reading times (10, 15, 20, 30 and 60 min) for detection of *Plasmodium *spp. in field study patients, Cambodia, 2011**

		**RDT diagnostic performance**			
		**Sensitivity (95%CI)**	**Specificity (95%CI)**	**PPV (95% CI)**	**NPV (95% CI)**
CareStart Malaria RDT	*Pf*	93.4% (87.4-97.1%)	98.6% (97.3-99.40%)	93.4% (87.4-97.10%)	98.6% (97.3-99.4%)
	*Non-Pf*	85.8% (80.7-90.0%)	99.1% (98.0-99.7%)	97.6% (94.4-99.2%)	94.5% (92.4-96.2%)
VIKIA Malaria Ag Pf/Pan RDT - Reading time 10 min.	*Pf*	89.7% (82.6-94.6%)	99.3% (98.3-99.8%)	96.3% (90.8-99.0%)	97.9% (96.4-98.9%)
	*Non-Pf*	61.5% (54.9-67.8%)	99.5% (98.5-99.9%)	97.9% (94.1-99.6%)	86.4% (83.6-89.0%)
VIKIA Malaria Ag Pf/Pan RDT - Reading time 15 min.	*Pf*	92.5% (86.2-96.5%)	99.1% (98.0-99.7%)	95.7% (90.2-98.6%)	98.4% (97.1-99.3%)
	*Non-Pf*	77.0% (71.1-82.2%)	99.5% (98.5-99.9%)	98.4% (95.3-99.7%)	91.4% (88.9-93.5%)
VIKIA Malaria Ag Pf/Pan RDT - Reading time 20 min.	*Pf*	93.4% (87.4-97.1%)	98.6% (97.3-99.40%)	93.4% (87.4-97.10%)	98.6% (97.3-99.4%)
	*Non-Pf*	81.1% (75.5-85.9%)	98.9% (97.7-99.6%)	96.9% (93.4-98.9%)	92.8% (90.5-94.7%)
VIKIA Malaria Ag Pf/Pan RDT - Reading time 30 min.	*Pf*	93.4% (87.4-97.1%)	98.6% (97.3-99.40%)	93.4% (87.4-97.10%)	98.6% (97.3-99.4%)
	*Non-Pf*	82.8% (77.4-87.4%)	98.9% (97.7-99.6%)	97.0% (93.6-98.9%)	93.4% (91.2-95.3%)
VIKIA Malaria Ag Pf/Pan RDT - Reading time 60 min.	*Pf*	94.2% (88.4-97.6%)	98.1% (96.6-99.0%)	91.2% (84.8-95.5%)	98.8% (97.5-99.5%)
	*Non-Pf*	82.7% (77.3-87.4%)	98.9% (97.7-99.6%)	97.0% (93.5-98.9%)	93.4% (91.1-95.2%)

Pf: P. falciparum; Non-Pf: Plasmodium non-falciparum.

PPV: Positive predictive value; NPV: Negative predictive value.

**Table 6:** **Diagnostic performance of the CareStart Malaria™ test and the VIKIA Malaria Ag Pf/Pan™ test at several reading times (10, 15, 20, 30 and 60 minutes) for different levels of *Plasmodium *spp. parasitaemia, Cambodia, 2011**

**Parasitaemia/μl of blood**		**CareStart Malaria RDT**		**VIKIA Malaria Ag Pf/Pan RDT - Reading time 10 min.**		**VIKIA Malaria Ag Pf/Pan RDT - Reading time 15 min.**	
		***Pf***	***Non-Pf***	***Pf***	***Non-Pf***	***Pf***	***Non-Pf***
Sub-microscopic	Sensitivity	16.7%	0.0%	16.7%	0.0%	16.7%	0.0%
< 100	Sensitivity	75.0%	68.7%	75.0%	46.7%	75.0%	50.0%
101-500	Sensitivity	71.4%	80.9%	57.1%	36.4%	57.1%	45.5%
501-1,000	Sensitivity	100.0%	72.7%	90.0%	36.4%	100.0%	72.7%
1,001-5,000	Sensitivity	100.0%	100.0%	100.0%	72.0%	100.0%	88.2%
5,001-10,000	Sensitivity	100.0%	100.0%	90.5%	74.3%	100.0%	92.1%
10,001-50,000	Sensitivity	100.0%	100.0%	100.0%	77.9%	100.0%	98.5%
50,001-100,000	Sensitivity	100.0%	100.0%	100.0%	100.0%	100.0%	100.0%
> 100,000	Sensitivity	100.0%	-	95.5%	-	100.0%	-
Parasitaemia/μl of blood		VIKIA Malaria Ag Pf/Pan RDT - Reading time 20 min.		VIKIA Malaria Ag Pf/Pan RDT - Reading time 30 min.		VIKIA Malaria Ag Pf/Pan RDT - Reading time 60 min.	
		*Pf*	*Non-Pf*	*Pf*	*Non-Pf*	*Pf*	*Non-Pf*
Sub-microscopic	Sensitivity	16.7%	0.0%	16.7%	0.0%	16.7%	0.0%
< 100	Sensitivity	75.0%	53.3%	75.0%	53.3%	75.0%	56.2%
101-500	Sensitivity	71.4%	57.1%	71.4%	61.9%	85.7%	61.9%
501-1,000	Sensitivity	100.0%	72.7%	100.0%	81.8%	100.0%	81.8%
1,001-5,000	Sensitivity	100.0%	94.0%	100.0%	98.0%	100.0%	98.0%
5,001-10,000	Sensitivity	100.0%	97.4%	100.0%	97.4%	100.0%	97.4%
10,001-50,000	Sensitivity	100.0%	100.0%	100.0%	100.0%	100.0%	100.0%
50,001-100,000	Sensitivity	100.0%	100.0%	100.0%	100.0%	100.0%	100.0%
> 100,000	Sensitivity	100.0%	-	100.0%	-	100.0%	-

Pf: P. falciparum; Non-Pf: Plasmodium non-falciparum.

## Discussion

This study reports the first evaluation of the performance of the VIKIA Malaria Ag Pf/Pan™, a new immunochromatographic test developed by IMACCESS®, using a combination of microscopy and real-time PCR as reference methods for classification of samples. This approach had the advantage of combining the high sensitivity (especially at low parasite density) and specificity (to correctly identify the parasite species) of real-time PCR, with the possibility of estimating parasite density by microscopy. As expected, more positive samples were found using real-time PCR than microscopy (+ 8.5%, 38.9% *vs* 42.2%), CareStart Malaria™ test (+9.7%, 38.1% *vs* 42.2%) or VIKIA Malaria Ag Pf/Pan™ test (from +10.4% for 60 min reading time to +27.0% for 10 min reading time, 30.8% and 37.8%, respectively *vs* 42.2%). A similar trend was also found for a greater sensitivity of microscopy by expert microscopists than malaria RDTs: 2.0% more positive samples by microscopy than CareStart Malaria™ test (38.8% *vs* 38.1%) and 2.8% (60 min reading time) to 20.8% (10 min reading time) more than with the VIKIA Malaria Ag Pf/Pan™ test (38.8% *vs* 37.8% and 30.8%, respectively).

The sensitivity (and the frequency of false negatives) of the VIKIA Malaria Ag Pf/Pan™ test increased with reading time, from 89.7% for *P. falciparum* detection and 61.5% for non-*P. falciparum* detection (10 min) to 94.2% for *P. falciparum* detection and 82.7% for non-*P. falciparum* detection (60 min). The specificity (and the frequency of false positives) decreased with reading time from 99.3% for *P. falciparum* detection and 99.5% for non-*P. falciparum* detection (10 min) to 98.1% for *P. falciparum* detection and 98.9% for non-*P. falciparum* detection (60 min).

The best performances of the VIKIA Malaria Ag Pf/Pan™ test for both *P. falciparum* and non-*P. falciparum* detection were observed at 20–30 min (sensitivity: 93.4% for *P. falciparum* and 81.1%-82.8% for non-*P. falciparum*; specificity: 98.6% for *P. falciparum* and 98.9% for non-*P. falciparum*). Using these reading times, the performance of the VIKIA Malaria Ag Pf/Pan™ test was similar to those of the CareStart Malaria™ test or those found in previous studies [21-26].

Moreover, as has been previously reported, the sensitivities of both malaria RDT decreased with the level of the parasitaemia [23,26-30]: for *P. falciparum*, the sensitivity of the both tests started to decrease at levels of parasitaemia < 500 parasites/μL (100% above 500 parasites/μL) and for non-*P. falciparum*, the sensitivity of the CareStart Malaria™ test and the VIKIA Malaria Ag Pf/Pan™ test started to decrease below 1,000 parasites/μL or below 10,000 parasites/μL, respectively. False negative results for both malaria RDT were only found in *P. falciparum* samples containing <250 parasites/μL and in non-*P. falciparum* samples with <1,000 parasites/μL.

According to WHO recommendations for RDT performances [31], sensitivity of the VIKIA Malaria Ag Pf/Pan™ test (except when the time reading was 10 min) was greater than 95%, excluding samples with a parasitaemia <100 parasites/μL. As observed for the CareStart Malaria™ test, the VIKIA Malaria Ag Pf/Pan™ test was easy to use and interpret and simple to store with no cold chain requirement.

## Conclusions

In conclusion, this new RDT performs similarly well as other commercially available tests (and in particular the CareStart Malaria™ test, used as comparator), and conforms to WHO recommendations for RDT performance. It appears to be a satisfactory alternative tool for the diagnosis of malaria in endemic areas.

## Competing interests

The authors declare that they have no competing interests.

## Authors’ contributions

MC, SK and DM contributed to the design and coordination of the study, assisted with data entry and interpretation and prepared the manuscript. SK supervised the field study. NK, SC, SS, DD and LC were involved in laboratory work. MC, SK, CN and DM helped to write the manuscript and gave constructive advice. All authors read and approved the final manuscript.
